# The benefit of active management in true knot of the umbilical cord: a retrospective study

**DOI:** 10.1007/s00404-024-07568-1

**Published:** 2024-06-03

**Authors:** Tal Weissbach, Shir Lev, Yonatan Back, Abeer Massarwa, Raanan Meyer, Tal Elkan Miller, Alina Weissmann-Brenner, Boaz Weisz, Shali Mazaki-Tovi, Eran Kassif

**Affiliations:** 1 Department of Obstetrics and Gynecology, Sheba Medical Center, Tel-Hashomer, Israel; 2 Sackler School of Medicine, Tel-Aviv University, Tel-Aviv, Israel; 3 Department of Obstetrics and Gynecology, Edith Wolfson Medical Center, Holon, Israel; 4 Division of Minimally Invasive Gynecology, Cedars Sinai Medical Central, Los Angeles, CA USA; 5 The Bornstein Talpiot Medical Leadreship Program, Sheba Medical Center, Tel Hashomer, Ramat-Gan Israel

**Keywords:** True knot of the umbilical cord, Prenatal diagnosis, Neonatal asphyxia, Non-reassuring fetal heart rate, Fetal death

## Abstract

**Purpose:**

To compare perinatal outcomes between active and routine management in true knot of the umbilical cord (TKUC).

**Methods:**

A retrospective study of singletons born beyond 22 ^6/7^ weeks with TKUC. Active management included weekly fetal heart rate monitoring(FHRM) ≥ 30 weeks and labor induction at 36–37 weeks. Outcomes in active and routine management were compared, including composite asphyxia-related adverse outcome, fetal death, labor induction, Cesarean section (CS) or Instrumental delivery due to non-reassuring fetal heart rate (NRFHR), Apgar_5_ score < 7, cord Ph < 7, neonatal intensive care unit (NICU) admission and more.

**Results:**

The Active (n = 59) and Routine (n = 1091) Management groups demonstrated similar rates of composite asphyxia-related adverse outcome (16.9% vs 16.8%, p = 0.97). Active Management resulted in higher rates of labor induction < 37 weeks (22% vs 1.7%, p < 0.001), CS (37.3% vs 19.2%, p = 0.003) and NICU admissions (13.6% vs 3%, p < 0.001). Fetal death occurred exclusively in the Routine Management group (1.8% vs 0%, p = 0.6).

**Conclusion:**

Compared with routine management, weekly FHRM and labor induction between 36 and 37 weeks in TKUC do not appear to reduce neonatal asphyxia. In its current form, active management is associated with higher rates of CS, induced prematurity and NICU admissions. Labor induction before 37 weeks should be avoided.

## What does this study add to the clinical work?

**Table:** 

This is the first comparative study to evaluate the benefit of weekly fetal monitoring and early labor induction as an active management strategy in umbilical-cord knot.	
Although there is some indication that active management may decrease fetal death, it does not appear to reduce neonatal asphyxia and is associated with more caesarean sections and induced prematurity.	

## Introduction

True knot of the umbilical cord (TKUC) is estimated to occur in 1–1.25% of singleton pregnancies [1–4]. Prenatal detection of TKUC was first described in 1991 by Collins et al. [5]. Although TKUC does not appear to impact long-term neurological outcome in exposed offspring [6], it has been associated with multiple adverse perinatal outcome including fetal death [1–3, 7, 8], non-reassuring fetal heart rate (NRFHR) [3, 7, 9], NRFHR-indicated Cesarean section (CS) [1, 7, 9, 10], meconium-stained amniotic fluid [3, 10], lower Apgar scores [2, 9, 10] and more [1–3, 7–10]. Beyond 37 weeks of gestation, the risk of fetal death is tenfold greater than that of singleton pregnancies without a TKUC [1]. Despite the higher rate of multiple adverse perinatal outcomes associated with TKUC, effective prenatal management strategies have not yet been investigated. This is probably because until recently, prenatal detection of TKUC was rare and inaccurate [7, 9, 11–14]. With advancements in ultrasound technology, it has recently been shown that TKUC can be accurately diagnosed prenatally, reaching a 96.4% detection rate when the umbilical cord is actively scanned [15]. Thus, the opportunity to assess management strategies has become available. The aim of this study was to compare perinatal outcomes between TKUC patients receiving active management, consisting of weekly fetal heart rate monitoring (FHRM) and labor induction between 36–37 weeks, and those receiving routine management.

## Materials and methods

**Fig. 1:**
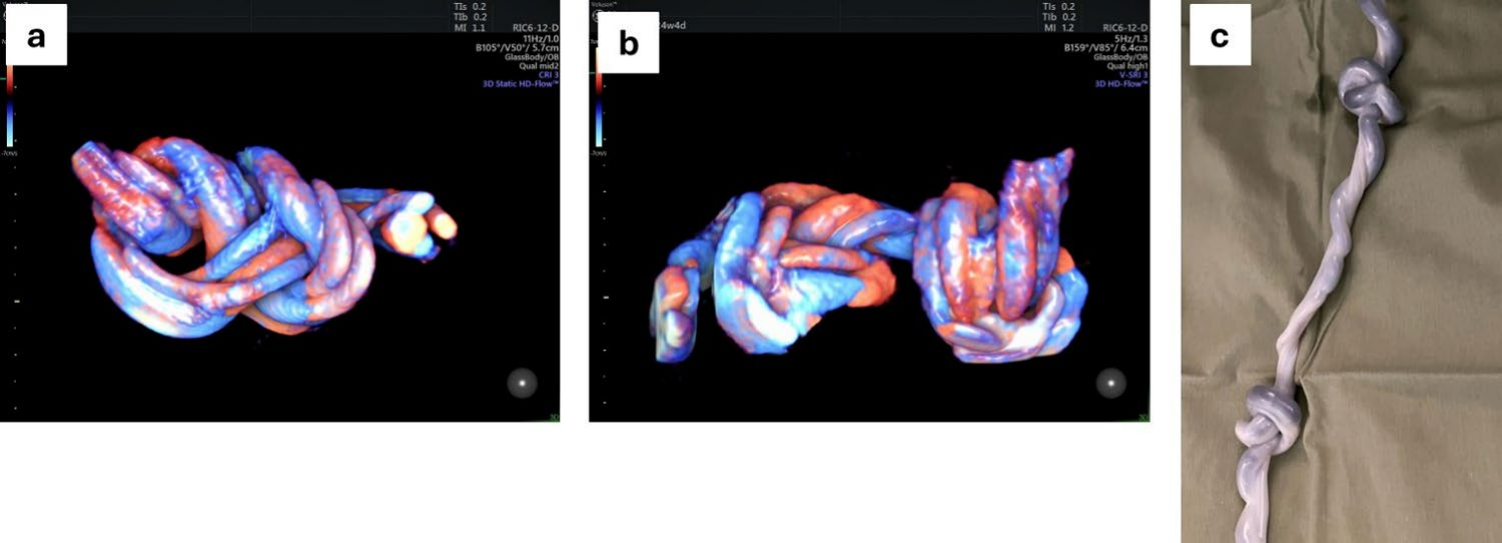
Prenatal and postnatal images of true knot of cord** a** 3D ultrasound demonstration of a single knot in the umbilical cord** b** 3D ultrasound demonstration of a double knot in the umbilical cord** c** postnatal image of a double knot in the umbilical cord

This was a retrospective study of all singletons born at a single tertiary center and diagnosed postnatally with TKUC between 2011 and 2022. Some of the patients in the current study have been reported on in 2 previous studies addressing different aspects of TKUC which were conducted at the same center [1, 15]. Singleton pregnancies that delivered livebirths and stillbirths beyond 22 ^6/7^ weeks of gestation, which were diagnosed postnatally with a TKUC, were included. High-order pregnancies and pregnancies terminated were excluded. The cohort was divided into 2 groups: (1) prenatally detected TKUC patients (Fig. 1) receiving active management (Active Management group) and (2) prenatally undetected TKUC patients receiving routine management (Routine Management group). The local departmental active management protocol included the following: 1. weekly FHRM from 30 weeks of gestation, 2. a growth scan at approximately 32 weeks of gestation and 3. labor induction between 36–37 weeks of gestation, or before, when fetal distress was suspected. Cases that were diagnosed at term were induced upon detection. Routine management included 1. a growth scan at approximately 32 weeks of gestation and 2. fetal well-being assessments beginning at 40 weeks of gestation, including growth assessment, FHRM and biophysical scans, which were conducted at the hospital every 2–3 days until delivery. Labor induction was recommended at 41.6 weeks or earlier in patients who presented other maternal/fetal indications.

The background maternal characteristics, including maternal age, parity, body mass index (BMI), previous cesarean deliveries and trial of labor after cesarean section (TOLAC), were compared between the groups. The perinatal outcomes compared included gestational age at delivery, birthweight, mode of delivery, rates of preterm birth (< 37 weeks), fetal death, labor induction, NRFHR [16], operative vaginal/CS delivery due to NRFHR, meconium-stained amniotic fluid (MSAF), 5-min Apgar (Apgar_5_) score < 7, umbilical cord pH < 7 (preferably arterial, venous in the absence of arterial), neonatal asphyxia, hypoxic ischemic encephalopathy (HIE), mechanical ventilation, NICU admission and neonatal death before discharge.

A composite asphyxia-related outcome, including fetal death, operative vaginal/CS delivery due to NRFHR, Apgar_5_ score < 7, umbilical cord pH < 7, neonatal asphyxia, HIE, mechanical ventilation, NICU admission and neonatal death before discharge, was compared between the groups.

### Definitions [17]

*Neonatal asphyxia* was defined as organ impairment presenting immediately after birth caused by compromised tissue oxygenation resulting from a suspected prenatal hypoxic event, characterized by NRFHR, a low APGAR score or a low cord Ph.

*Hypoxic-ischemic encephalopathy* was defined as neurological impairment appearing immediately after birth resulting from suspected asphyxia with one or more of the following presentations: low Apgar score (< 7), seizures and reduced tone, decreased reflexes, depressed respiratory effort or decreased level of consciousness.

The study protocol was approved by the Institutional Review Board (approval number 5345–18-SMC).

### Statistical analysis

The normality of the data was tested using the Shapiro‒Wilk or Kolmogorov‒Smirnov tests. Continuous variables are presented as medians and interquartile ranges (IQRs), and categorical variables are presented as numbers (%). Comparisons between unrelated variables were conducted with Student's t test or the Mann–Whitney U test, as appropriate. The chi-square test and Fisher's exact test were used for comparisons between categorical variables. Statistical significance was accepted at p < 0.05. Statistical analyses were conducted using the IBM Statistical Package for the Social Sciences (IBM SPSS v.25; IBM Corporation Inc., Armonk, NY, USA).

## Results

During the study period, there were 121,435 singleton births at our center, 1158 of which were postnatally diagnosed with a TKUC, yielding an incidence of 0.95%. Four cases of pregnancy termination and 4 cases of twin pregnancy were excluded, leaving 1150 cases available for analysis. The Active Management group included 59 patients, and the Routine Management group included 1091 patients. The median gestational age at the time of knot detection was 23 ^4/7^ weeks (IQR 22–30 ^6/7^). Over one-third (39%) of the cases were detected on a targeted scan for various anomalies or conditions, such as FGR, single umbilical artery, placenta accreta, previous IUFD, NRFHR, liver calcifications, increased HCG, echogenic bowel, aortic stenosis, genetic abnormality, placental abruption and placental lesions, while the rest were detected on routine anomaly or biophysical scans. Table 1 displays a comparison of background characteristics of the groups. There were no statistically significant differences between the groups regarding maternal age and BMI, rates of nulliparity, previous CS or trial of labor after TOLAC.

**Table 1:** Comparison of Background Characteristics

	Active management, N = 59	Routine management, N = 1091	P value
Age	34 (31–36)	34 (30–37)	0.27
Nulliparity	16.9% (10/59)	23.2% (253/1091)	0.27
BMI	27.3 (25–29.8)	28.1 (25.4–31.6)	0.07
Previous CS	18.6% (11/59)	13.8% (151/1091)	0.3
TOLAC*	20% (1/5)	64.3% (72/112)	0.06
GA at Knot Detection (Weeks)	23.4 (22–30.6)	N/A	

Data are presented as Median (Interquartile Range) or %(N)

*BMI* body mass index, CS cesarean section, *TOLAC* trial of labor after cesarean, *GA* gestational age

^*^Among patients after 1 cesarean section

### Perinatal adverse outcome

A comparison of the perinatal outcomes of the entire cohort is presented in Table 2. Both groups demonstrated a similar rate of composite asphyxia-related outcome (16.9% vs 16.8%, p = 0.97). There were no statistically significant differences detected in the individual perinatal outcomes between the groups, except for MSAF, which was lower in the active management group (1.7% vs 18%, p < 0.001), and NICU admissions, especially prematurity-related NICU admissions, which were greater in the active management group (13.6% vs 3%, p < 0.001 and 8.5% vs 1.7%, p = 0.005, respectively). Fetal death occurred exclusively in the Routine Management group (1.8%, 20/1091), but this difference did not reach statistical significance (p = 0.62).

**Table 2:** Comparison of perinatal and asphyxia-related outcomes in all patients

	Active Management (59)	Routine Management (1091)	P value
GA at Delivery	37.1 (36.6–37.3)	39.2 (38.2–40.2)	** < 0.0001**
Birthweight	2822 (2478–3085)	3260 (2942–3550)	** < 0.0001**
FGR	13.6% (8/59)	6.9% (75/1091)	0.07
Fetal Death*	0%	1.8% (20/1091)	0.6
NRFHR**	20.3% (12/59)	28.9% (310/1072)	0.16
CS/Operative Vaginal Delivery due to NRFHR**	10.2% (6/59)	13.2% (142/1072)	0.5
Meconium-Stained Amniotic Fluid	1.7% (1/59)	18% (196/1091)	**0.001**
Prematurity***	27.1% (16/59)	5.3% (57/1071)	** < 0.001**
Induced Prematurity***	22% (13/59)	1.7% (18/1071)	** < 0.001**
Indication for Induced Prematurity			
Maternal/Fetal Distress^a^	61.5% (8/13)	72.2% (13/18)	**0.7**
Risk Factors/Maternal Request^b^	38.5% (5/13)	27.8% (5/18)	
Apgar_5 min_ < 7***	3.4% (2/59)	0.7% (8/1071)	0.09
Cord Ph < 7***	0%	0.3% (3/1003)	1
Neonatal Asphyxia***	1.7% (1/59)	0.5% (5/1071)	0.28
Hypoxic Ischemic Encephalopathy***	0%	0.4% (4/1071)	1
Mechanical Ventilation***	3.4% (2/59)	1.3% (14/1071)	0.2
NICU Admission***	13.6% (8/59)	3% (32/1071)	** < 0.001**
NICU Admission due to Prematurity***	8.5% (5/59)	1.7% (18/1071)	**0.005**
NICU Admission due to Asphyxia***	0%	0.5% (5/1071)	1
Neonatal Death Before Discharge	0%	0%	N/A
Composite Asphyxia-Related Outcome****	16.9% (10/59)	16.8% (183/1091)	0.97

Bold emphasized statistical significance

Data are presented as Median (Interquartile Range) or %(N)

*GA* gestational age, *FGR* fetal growth restriction, *NVD* normal vaginal delivery, *CS* cesarean section, *NRFHR* non-reassuring fetal heart rate

^*^One case occurred intrapartum, during an emergency cesarean section

^**^Not including 19 cases of antepartum fetal death

^***^Not including 20 cases of fetal death

^****^Including fetal death, operative vaginal/CS delivery due to NRFHR, Apgar_5_ score < 7, umbilical cord pH < 7, neonatal asphyxia, hypoxic ischemic encephalopathy, mechanical ventilation, NICU admission and neonatal death before discharge

a- placental abruption (3), suspected uterine dehiscence/rupture (3), NRFHR (12), maternal variceal bleeding (1), preeclampsia (2)

b- true knot (4), placenta accreta (1), maternal request (2), previous fetal death (2), previous placental abruption (1)

As expected, the Active Management group delivered earlier and at lower median birthweights than did the Routine Management group (37 ^1/7^ vs 39 ^2/7^ weeks of gestation, 2822 g versus 3260 g, p < 0.0001, for both). There was a higher rate of prematurity (27.1% vs 5.3%, p < 0.001) and induced prematurity (81.3% vs 31.6%, p < 0.001) in the Active Management group. There was a similar dispersion of iatrogenic prematurity indication, with most cases induced for maternal/fetal distress in both groups (72.2% vs 61.5%, p 0.7%).

### Clinical significance of meconium-stained amniotic fluid

The rate of MSAF was found to be significantly lower in the active management group. Since both fetal distress and advanced gestational age at the time of delivery are known to be associated with MSAF [18], collinearity was suspected. Binary logistic regression was performed and included the following variables: gestational age at the time of delivery, study group assignment (Active/Routine Management) and NRFHR. Of these, only gestational age at delivery and NRFHR were found to be independently associated with MSAF in the current cohort.

### Delivery outcomes

Delivery outcomes are presented in Table 3. Significant differences were found regarding the mode of delivery between the groups. There was a higher rate of CS and a lower rate of normal vaginal delivery in the Active than in the Routine Management group (37.3% vs 19.2% and 55.9% vs 74.4%, respectively, p = 0.003). There was a higher rate of primary CS without trial of labor (TOL) in the Active Management group (27.1% vs 11%, p < 0.0001). Accordingly, there was a lower rate of TOL in the Active Management group (72.9% vs 89%, p < 0.0001).

**Table 3:** Delivery outcomes for entire cohort and among trial of labor

	Active Management (59)	Routine Management* (1072)	P value
Mode of Delivery*			
NVD	55.9% (33/59)	74.4% (798/1072)	**0.003**
Operative Vaginal	6.8% (4/59)	6.3% (68/1072)	
CS	37.3% (22/59)	19.2% (206/1072)	
Primary Cesarean** ^±^	27.1% (16/59)	11% (118/1072)	** < 0.001**
Maternal Request Primary Cesarean	3.4% (2/59)	0.7% (8/1072)	0.09
Trial of Labor	72.9% (43/59)	89% (954/1072)	** < 0.001**
Labor Induction among TOL	53.5% (23/43)	13.2% (126/954)	** < 0.001**
Vaginal Delivery of Any Type	62.7% (37/59)	80.8% (867/1072)	** < 0.001**
Mode of Delivery among TOL			
NVD	76.7% (33/43)	83.6% (798/954)	0.48
Operative Vaginal	9.3% (4/43)	7.1% (68/954)	
Cesarean Section	14% (6/43)	9.2% (88/954)	
Vaginal Delivery of Any Type among TOL	86% (37/43)	90.8% (866/954)	0.3
CS due to NRFHR among TOL	0% (0/43)	6.1% (58/954)	0.17
CS/Operative Vaginal Delivery due to NRFHR	4.7% (2/43)	12.2% (116/954)	0.14

Bold emphasized statistical significance

Data are presented as Median (Interquartile Range) or %(N)

*NVD* normal vaginal delivery, *CS* cesarean section, *TOL* trial of labor, *NRFHR* non-reassuring fetal heart rate

^*^Not including 19 antepartum fetal demise cases

^**^CS indications: Malpresentation, previous cesarean section, maternal request, severe IUGR, macrosomia, suspected placenta accreta, and suspected cephalopelvic disproportion

± Birth by Cesarean with no trial of labor

Among patients attempting TOL, there were similar rates of vaginal deliveries (86% vs 90.8%, p = 0.3), including spontaneous vaginal deliveries (76.7% vs 83.6%, p = 0.48). Notably, the Active Management group presented a nonsignificant trend toward lower rates of CS due to NRFHR and of CS/instrumental vaginal delivery due to NRFHR than did the Routine Management group (0% vs 6.1%, p = 0.17 and 4.7% vs 12.2%, p = 0.14).

### Fetal death characteristics

The characteristics of the 20 cases o fetal death are presented in Table 4. The median and interquartile range of gestational age at fetal death was 36.5 ^5/7^ weeks (33–38 ^2/7^). Half of the fetal deaths occurred at term, and 95% (19/20) occurred after 30 weeks of gestation. A quarter of the fetuses were growth restricted, significantly more than among TKUC survivors (25% vs 6.8%, p = 0.01). Fetal well-being assessment, including FHRM and biophysical profile, was performed in half of the fetal death cases in the preceding week. A reassuring fetal status was determined in 90% (9/10) of the fetal death cases assessed by FHRM. One case of fetal death was preceded by recurrent moderate decelerations on FHRM. Despite an emergency CS, the fetus was stillborn and did not respond to cardio-respiratory resuscitation.

**Table 4:** Characteristics of fetal deaths

	Fetal death (20)
Maternal Age	33.5 (27.5–37.5)
Maternal BMI	29 (25.5–34)
FGR*	25% (5/20)
Suspected Anomalies**	11.8% (2/17)
Non-Reassuring Fetal Status before Fetal Demise	5% (1/20)
Proven Reassuring Fetal Status in the Preceding Week	45% (9/20)
GA at Fetal Death, Median (IQR)	36.5 (33–38.2)
Range	23.5–41.6
GA at Fetal Death	
≥ 37 weeks	50% (10)
30–36.6	45% (9)
< 30 weeks	5% (1 at 23.5 weeks)

Data are presented as Median (Interquartile Range) or %(N)

*BMI* Body Mass Index, *FGR *fetal growth restriction, Cesarean, GA gestational age

^*^There was a higher rate of IUGR among fetal death cases than among live births (25% vs 6.8%, p = 0.01)

^**^Three patients did not undergo anomaly scans. The suspected anomalies were PRUV and aortic coarctation

## Discussion

This is the first study to evaluate the benefit of active management of prenatally detected TKUC, consisting of weekly fetal monitoring from 30 weeks and labor induction between 36 and 37 weeks. Previous studies have investigated other aspects of TKUC, including associated risk factors, perinatal outcomes and prenatal detection [1–4, 7–9, 11, 13–15, 19–23]. TKUC was found to be associated with fetal death [1–3, 7, 8], NRFHR [3, 7, 9], CS due to NRFHR [1, 7, 9, 10], low Apgar score [2, 8, 10], and meconium-stained amniotic fluid [3, 10]. The principal findings of the current study are that active management of TKUC confers a comparable rate of composite asphyxia-related outcome to that of routine management. Furthermore, active management in its current form is associated with increased rates of CS, labor induction, prematurity, induced prematurity, and NICU admissions, especially due to prematurity. These findings question the justification of active management in its current form.

The main purpose of active management is to prevent fetal death and asphyxia. Although rare, it has been shown by Weissmann-Brenner et al. to be more common in pregnancies affected by TKUC, with a tenfold increased risk of fetal death from 37 weeks of gestation compared to the general fetal population, reaching 1% at term [1]. In accordance with this study, half of the fetal deaths in the current study occurred at term, supporting the rationale for labor induction at 37 weeks as a preventative measure. The fact that all fetal deaths occurred exclusively in the Routine Management group implies that active management may prove to be beneficial; however, a larger cohort is needed to statistically confirm this observation.

The benefit of weekly fetal well-being assessment is questionable since fetal death seems to be acute and unpredictable. In the present study, fetal death occurred within a week of a reassuring fetal assessment in nearly half of the patients, implying that FHRM is ineffective for predicting fetal death in TKUC. Moreover, intrapartum FHRM has been shown to be unaffected by TKUC, presenting similar FHRM patterns in pregnancies with and without TKUC [2, 21]. Therefore, the evidence does not support intermittent FHRM as an effective measure for preventing fetal death.

Interestingly, our study revealed a lower rate of MSAF in the active management group. Previous studies have shown higher rates of MSAF in patients with TKUC than in controls. MSAF has been proven to be associated with both fetal distress and advanced gestational age [18]. Similarly, multiple regression in the current study revealed that MSAF was independently associated with both gestational age at delivery and NRFHR but not with active/routine management group assignment. Whether this reduction in MSAF may aid in preventing the rare occurrence of Meconium Aspiration Syndrome should be further assessed in a large cohort. None of the patients in the current study experienced this dire outcome.

Unfortunately, active management appears to have had a negative impact on delivery outcomes. First, there were fewer attempts at TOL, lower rates of vaginal delivery and higher rates of primary CS, without a trial of labor. This may be partially due to maternal anxiety caused by the knowledge of an existing TKUC, as reflected in the nonsignificant trend of a higher rate of CS at maternal request. The effect of maternal anxiety in TKUC on the choice of CS as a mode of delivery was also described in a previous study by Bohiltea et al. [9]. In addition to maternal anxiety, patients in the Active Management group with a history of a single CS might have chosen an elective re-CS instead of attempting labor induction to avoid uterine rupture [24, 25]. Nevertheless, our data show that patients receiving active management and attempting TOL had equally high rates of successful vaginal deliveries as patients receiving routine management (86% vs 90.8%, p 0.3). Therefore, patients with prenatally detected TKUC should be encouraged to attempt TOL. Patients with a history of a single low segment CS should be counselled on the risk of uterine rupture during labor induction in TOLAC which has been shown to be slightly increased compared to spontaneous labor in TOLAC [26, 27].

A second disadvantage of active management was the increased rate of prematurity, especially induced prematurity, which led to increased rates of adverse neonatal outcomes, including increased NICU admissions due to prematurity. Fortunately, some cases of induced prematurity can be prevented by avoiding preterm labor induction due to TKUC in the presence of reassuring fetal well-being, as occurred in 30.8% of the cases of induced preterm birth in the current study. This approach is supported by the observation of Weissmann-Brenner et al. of a tenfold increased risk of fetal death from 37 weeks of gestation in TKUC compared to the general fetal population. Before term, there was no statistically significant difference in the rate of fetal death [1].

The clinical value of routinely scanning the entire length of the umbilical cord is questionable. This depends on the benefit of an intervention to prevent fetal death and asphyxia associated with TKUC. Currently, there is no evidence to support this practice. Should a sufficiently powered study prove an advantage for some form of active management, then a routine scan of the umbilical cord would be justified. The current study presents preliminary data to support a larger future study. Technically, scanning the entire length of the cord at mid-trimester has been shown to be feasible with an accuracy of 96.4% [15].

The current study is not without limitations. First, due to the relatively small number of prenatally detected knots and the rarity of fetal deaths, this study was underpowered to evaluate this outcome. At least 300 active management cases are needed to prove a statistically significant difference in fetal death at a power of 80%. Furthermore, a larger cohort might reveal other statistically significant differences, which in the current study appear as trends, such as a lower rate of NRFHR and CS due to NRFHR. Second, the retrospective design of the study has inherent methodological flaws, including a selection bias of high-risk patients in the Active Management group, who were more frequently scanned for various indications, facilitating the detection of TKUC in this population. Another inherent limitation of the retrospective design was the nonuniform management of both groups, since department policy slightly evolved over the 11-year period of the study. For example, in earlier years, patients diagnosed with TKUC were induced at 36 weeks, and over the years, induction was postponed to 37 weeks. Ideally, in order to optimally evaluate the benefit of active management in TKUC, a randomized control trial randomizing patients with a prenatally detected TKUC to routine and active management should be performed. However, considering the evidence of increased fetal death [2–4, 7, 8], especially at term [1], it may be regarded unethical and pose legal risks to intentionally provide routine management in pregnancies with a known TKUC.

This study has several strengths worth mentioning. First, this is a novel study evaluating the impact of active management in TKUC, a subject that has never been studied before. Second, the study comprises the largest cohort of prenatally diagnosed TKUCs in the published literature, offering insights that may improve the perinatal outcome in pregnancies diagnosed with a TKUC, such as avoiding unnecessary induced prematurity and unindicated CS.

To conclude, compared with routine management, weekly FHRM and labor induction at 36–37 weeks in patients with TKUC do not appear to reduce neonatal asphyxia. In its current form, active management is associated with higher rates of cesarean deliveries, induced prematurity, and NICU admissions. Based on these observations, labor induction before 37 weeks should be avoided. In the absence of a contraindication, patients can be encouraged to attempt vaginal delivery. Although there is a certain indication that active management may reduce fetal death a larger cohort is needed to determine a true advantage.

## Data Availability

The data that support the findings of this study are available on request from the corresponding author.
